# Dysfunction of Liver Receptor Homolog-1 in Decidua: Possible Relevance to the Pathogenesis of Preeclampsia

**DOI:** 10.1371/journal.pone.0145968

**Published:** 2015-12-30

**Authors:** Dongmei Zhang, Dong Cheng, Tao Liu, Yachao Zhang, Zi-Jiang Chen, Cong Zhang

**Affiliations:** 1 Center for Reproductive Medicine, Ren Ji Hospital, School of Medicine, Shanghai Jiao Tong University, Shanghai, China; 2 Shanghai Key Laboratory for Assisted Reproduction and Reproductive Genetics, Shanghai, China; 3 Shandong Center for Disease Control and Prevention, 16992 Jingshi Road, Ji’nan, Shandong, 250014, China; 4 Key Laboratory of Animal Resistance Research, College of Life Science, Shandong Normal University, 88 East Wenhua Road, Ji’nan, Shandong, China; Chinese Academy of Sciences, CHINA

## Abstract

Preeclampsia (PE) is a multisystem disorder unique to *Homo sapiens* that is known to cause maternal and perinatal mortality and morbidity. Between 5–7% of all pregnancies are affected by PE and it is responsible for approximately 50,000 maternal deaths annually. The pathogenesis of PE remains poorly understood. However, the results of this study indicated that insufficient decidualization plays a significant role. *NR5A1* and *NR5A2* are orphan members of the Ftz-F1 subfamily of nuclear receptors and are involved in mammal follicular development, female reproduction, steroidogenesis, and decidualization. The expression of *NR5A1* and *NR5A2* in the human decidua and their functions during decidualization were investigated using *in vitro* cultured cells by real-time PCR, immunohistochemistry, western blotting, and siRNA techniques. The results demonstrated that the levels of *NR5A2* mRNA and protein in the decidual tissues of women with PE were lower than those of normal pregnant women. However, the levels of *NR5A1* mRNA and protein did not significantly differ between groups. The expression of *NR5A2* was upregulated after *in vitro* decidualization, but the expression of *NR5A1* remained low and showed no difference compared with that of the control cells. Knocking down of *NR5A2* in human endometrial stromal cells (hESC) resulted in a significant reduction in their expression of decidualization markers (*IGFBP1* and *PRL*) and signaling pathway molecules (*WNT4* and *BMP2*) (*P* < 0.05). From these data, we concluded that *NR5A2* is pivotal for the decidualization of decidual tissues and cultured human endometrial stromal cells. Disorders of the endometrium in decidual tissues may be associated with the abnormal decidualization thought to cause PE.

## Introduction

Preeclampsia (PE) is characterized by the occurrence of hypertension and/or proteinuria after 20 weeks of gestation. It is a serious complication of the second half of pregnancy, labor, or the early period after delivery. PE is responsible for maternal and fetal morbidity and mortality affecting 5–7% of all pregnancies [1] and is responsible for 42% of all maternal deaths and 15% of all preterm deliveries [2]. Pregnant women with PE demonstrate increased blood pressure, proteinuria, edema, abnormal clotting, and liver and renal dysfunction. Fetal PE syndrome can manifest as preterm delivery, growth restriction, placental abruption, and fetal distress [3]. Furthermore, the long-term effects of PE can include cardiovascular complications for both the mother and the child.

PE is a multisystem disorder, and it is generally believed that PE is associated with incomplete remodeling of the uterine spiral arteries, deficient invasion of extravillous trophoblastic (EVT) cells into the decidua and myometrium [4, 5], deregulation of immunological response, abnormal production of inflammatory factors, and failure to regulate hormone, prostaglandin, and lipid metabolism [6]. In addition, defective decidualization may contribute to the compromised invasion of EVT cells in PE [7]. The superficial invasion of EVT cells and impaired spiral artery remodeling are hallmarks of PE. The invasion of EVT cells into the uterine tissues is of crucial importance for successful placental and fetal development and the progression of pregnancy. As a result, it is tightly temporally and spatially regulated. Despite decades of research, a full understanding of the pathogenesis of PE remains elusive.

One of the initial processes in human pregnancy is the attachment of the blastocyst to the uterine decidua. The EVT cells invade and proliferate into the uterine decidua to anchor the developing embryo to the uterus and establish an appropriate supply of nutrients and oxygen for the fetus [8, 9]. In humans, under the stimulation of progesterone, decidualization first begins in the endometrial stromal cells surrounding the spiral arteries of the uterus during the late secretory phase of the menstrual cycle [10]. At this time the endometrium begins to undergo remodeling in preparation for embryo implantation. Specifically, the endometrial stromal cells undergo a marked rearrangement of the intracellular architecture and begin to accumulate glycogen, initiating the secretion of various proteins, growth factors, and cytokines such as prolactin (PRL) and insulin-like growth factor binding protein 1 (IGFBP1). All of these changes accompany the morphological transition from stromal cells to larger, more rounded decidual cells that are essential to support embryo implantation [11, 12], control EVT cell invasion into the endometrial bed, and modulate the maternal immune response [13]. The decidualization persists and extends throughout the endometrium, leading to the formation of the pregnancy decidua with embryo implantation [10].

Steroidogenic factor-1 (*SF-1*; *NR5A1*) and liver receptor homolog-1 (*LRH-1*; *NR5A2*) are orphan members of the Ftz-F1 subfamily of nuclear receptors. Both of them have been demonstrated to be involved in the regulation of steroid metabolism and hormone synthesis [14]. *NR5A1* is evolutionarily closely related to *NR5A2* [15], and its expression is confined to steroidogenic tissues and the hypothalamo-pituitary-adrenal axis, where it is involved in the control of development, differentiation, steroidogenesis, and sexual determination of the fetus [16, 17]. *NR5A1* is also expressed in whole ovary including granulosa, thecal, luteal, and interstitial cells in immature and adult rodents [18, 19]. *NR5A1* binds to its consensus DNA sequence and activates the transcription of target genes [20, 21] such as aromatizing enzyme, luteinizing hormone, follicle-stimulating hormone, prolactin, gonadotropin releasing hormone receptor, corticotropin releasing hormone, and others. Zeitoun and Xue found that the mRNA and protein levels of *NR5A1* in endometriotic stromal cells were significantly higher than those in normal endometrial stromal cells [22, 23]. The aberrant expression of *NR5A1* in endometrial stromal cells may be related to the pathogenesis of endometriosis. *NR5A2* is expressed in tissues derived from the endoderm, including the intestine, liver, exocrine pancreas, and ovary. In these tissues, *NR5A2* plays a predominant role in development, reverse cholesterol transport, bile acid homeostasis, and steroidogenesis [14]. In the ovary, *NR5A2* is expressed by the granulosa cells of pre-ovulatory follicles and luteal cells of the newly formed corpus luteum in rodents [18, 19, 24], and is essential for ovulation [25]. Our previous study also illustrated that *NR5A2* is crucial for human decidualization, which is necessary for successful pregnancy, by regulating the expression of its target gene *WNT4* [14]. In addition, bone morphogenetic protein 2 (*BMP2*) is also involved in decidualization by regulating *WNT4* [26].

Maruyama et al. demonstrated that decidualization is crucial for embryo implantation and the maintenance of pregnancy [10]. Impaired decidual responses may cause a variety of endometrial and pregnancy disorders including infertility, recurrent miscarriages, uteroplacental dysfunction, endometriosis, endometrial cancer, and PE. Our research also found that the deficiency of decidualization caused by the knocking out of *NR5A2* induced a PE phenotype in mouse placenta [14]. The expression patterns of *NR5A1* and *NR5A2* in the decidual tissues of pregnant women, their exact roles in decidualization, and their relationship with the pathology of PE are hence the foci of this study. Immunohistochemistry, real time PCR, western blots, *in vitro* cell culture, and RNA interference were used to explore the expression of *NR5A1* and *NR5A2* and their relationships with PE.

## Materials and Methods

### Study population

This project was reviewed and approved by the Ethics Committee of Qilu Hospital, and written consent was obtained from all participants.

A total of 23 women with severe preeclampsia (SPE) and 23 women with normal pregnancy (NP) were recruited from the Qilu Hospital of Shandong University during the period from January 2014 to January 2015. SPE was diagnosed according to the regulations of the International Society for the Study of Hypertension in Pregnancy. Women with fetal malformations, chronic hypertension, renal disease, diabetes mellitus, and polycystic ovarian syndrome were excluded. Women with only one pregnancy were included. All the pregnancies were delivered by cesarean section. Decidual tissue was collected immediately after delivery and the decidua was obtained from the gauze used to scrub the uterus. The tissues were washed with sterilized water to remove blood and the samples were snap frozen in liquid nitrogen immediately after collection and were stored at -80°C until use.

### Immunohistochemistry to evaluate NR5A1 and NR5A2 in decidual tissues

Immunolocalization of NR5A1 and NR5A2 proteins was performed using cryosections (5 μm) as described previously [27]. The sections were fixed in acetone at -20°C for 10 min and then incubated in 0.3% (v/v) Triton™ X-100 in phosphate-buffered saline (PBS) (pH 7.2) for 20 min. Endogenous peroxidase activity was quenched by incubating the sections in 0.3% (v/v) hydrogen peroxide for 30 min. The sections were then washed with PBS, and the primary antibodies against NR5A1 (AP5106a, Abgent, San Diego, CA, USA) at 1:200 dilution and NR5A2 (SC-21132; Santa Cruz Biotechnology, Santa Cruz, CA, USA) at 1:100 dilution were applied at 4°C overnight. After washing with PBS for three times, the samples were incubated with peroxidase-conjugated rabbit anti-goat IgG secondary antibody for 1 h at room temperature. The colorimetric reactions were developed using a standard diaminobenzidine kit (ZLI-9033, ZSGB-BIO, Beijing, China). The sections were then counterstained with hematoxylin, dehydrated, mounted, and photographed using an ML2000 microscope (Olympus, Tokyo, Japan). Negative controls were incubated with non-immune serum instead of the primary antibody.

### Human endometrial stromal cell (hESC) culture, in vitro decidualization and transfection with siRNA

The hESC line was immortalized by the retroviral transfection of human telomerase (ATCC CRL-4003) as described by Krikun et al [28] and was a kind gift from Dr. Haibin Wang (Institute of Zoology, Chinese Academy of Sciences, Beijing, China). The cells were cultured in Dulbecco’s modified Eagle’s medium/F-12 without phenol red (Life Technologies Inc., Grand Island, NY, USA) containing 10% (v/v) fetal bovine serum (Biological Industries, Beit Haemek, Israel), 1% (v/v) insulin-transferrin-selenium solution (Invitrogen, Carlsbad, CA, USA), 5 × 10^−4^ g/L puromycin (Gibco, Grand Island, NY, USA), 5 × 10^4^ U/L penicillin, and 5 × 10^−2^ g/L streptomycin (Gibco).

To induce *in vitro* decidualization, the concentration of fetal bovine serum was reduced to 2% (v/v), the cells were treated with 10^−3^ mM medroxyprogesterone-17-acetate (MPA) (Sigma Chemical Co., St. Louis, MO, USA), and 0.5 mM N6, 2′-*O*-dibutyryladenosine cAMP sodium salt (db-cAMP) (Sigma Chemical Co.) for 7 days, while control samples were treated with 0.1% (v/v) ethanol. As for siRNA transfection, Lipofectamine RNAiMAX Reagent (Invitrogen) was mixed with 5 × 10^−5^ mM *NR5A2* siRNA (Invitrogen), or with a pool of non-targeting control siRNAs. The mixtures were then added to hESCs at 60–80% confluency in 6-well culture plates. After 24 h, the siRNA was removed and the cells were induced to decidualization for 4 days.

### RNA isolation and real-time PCR

Total RNA was isolated using an Animal Total RNA Isolation Kit (Foregene, Chengdu, China) according to the manufacturer’s instructions. Reverse-transcription of 1 μg of RNA was performed using the ReverTra Ace^®^ qPCR RT Kit (Toyobo, Osaka, Japan). Quantitative RT-PCR was performed using a Applied Biosystem qRT-PCR System (Life Technologies Inc.) under the following conditions: 3 min at 95°C; 40 cycles of 15 s at 95°C, 30 s at 60°C, 30 s at 72°C; and 5 min at 72°C. Melting-curve analyses were carried out to verify the product identities. PCR was performed in triplicate and was expressed relative to the abundance of endogenous *GAPDH* in the same sample. The primer sequences are given in S1 Table. The reaction efficiency was determined using LinRegPCR 11.0 software (Academic Medical Center, Amsterdam, Netherlands) and data were calculated relative to a calibrator sample using the ΔΔCt method [29, 30].

### Western blot analysis

Total protein was isolated from decidual tissue and hESCs using RIPA buffer (50 mM TRIS pH 7.4, 150 mM NaCl, 1% [v/v] Triton™ X-100, 1% [w/v] sodium deoxycholate, 0.1% [w/v] sodium dodecyl sulfate [SDS]) with protease inhibitors (Sigma Chemical Co.). The isolated proteins were quantified with an Enhanced BCA Protein Assay Kit (Thermo Fisher Scientific, Waltham, MA, USA). A solution containing 20 μg of protein was solubilized in a sample buffer consisting of 62.5 mM Tris- HCl pH 6.8, 2% (w/v) SDS, 25% (v/v) glycerol, 0.01% (w/v) bromophenol blue, and 5% (w/v) β-mercaptoethanol and boiled for 5 min at 100°C. The samples were then separated via 10% (v/v) SDS-polyacrylamide gel electrophoresis and transferred to a polyvinylidene fluoride membrane (GE Healthcare Life Sciences, Little Chalfont, Buckinghamshire, UK). The membrane was then blocked with 5% (w/v) nonfat milk powder at room temperature for 1 h followed by an incubation with primary antibodies against NR5A1 (1:500 dilution in PBS), NR5A2 (1:500 dilution), or β-actin (SC-47778, Santa Cruz Biotechnology; 1:5000 dilution) at 4°C overnight. The membranes were washed with Tris-buffered saline containing Tween^®^ 20 3 times and incubated with horseradish peroxidase-conjugated anti-rabbit IgG or anti-goat IgG antibody (1:5000 dilution) at room temperature for 1–2 h. The bands were detected after incubation with Immobilon™ Western Chemiluminescent HRP Substrate (Millipore, Billerica, CA, USA) using an Enhanced Chemiluminescence Western Blotting Detection System (Tanon Science & Technology Co., Ltd., Shanghai, China). Proteins from three separate western blot experiments were analyzed by QuantiScan software (Biosoft, Cambridge, UK). The relative densities of the detected bands were calculated by normalizing to the densities of bands for β-actin in the same blot.

### Statistical analysis

The data were analyzed using SPSS statistical software (SPSS Inc., Chicago, IL, USA). The differences between the two groups (SPE and NP) were analyzed by one-way analysis of variance and Student’s t-test. The data are represented as means ± SEM and all experiments were repeated at least three times. A value of *P* < 0.05 was regarded as statistically significant.

## Results

### Characteristics of the participants

The characteristics of the patients with SPE and the NP controls were analyzed (Table 1). No significant difference in maternal age between the NP and SPE groups was observed. The gestational age of the SPE group was significantly shorter than that of the NP group (*P* < 0.01). Correspondingly, the birth weight of newborns in the SPE group was also significantly lower than that of the NP group (*P* < 0.01). The systolic and diastolic blood pressures of subjects were significantly higher in the SPE group compared to those of the NP group (*P* < 0.01). The proteinuria of the SPE patients was more than 2+ by protein dipstick grading, while normal pregnant women had no proteinuria.

**Table 1:** Patient characteristics of women with severe PE (SPE) and the normal controls (NP).

	NP (n = 23)	SPE (n = 23)	P
**Maternal age (years)**	28.67±0.532	29.17±1.537	0.6542
**Gestational age (weeks)**	39.22 ± 0.462	36.87 ± 0.769	0.0002
**Birth weight (g)**	3635 ± 118.5	2703 ± 189	< 0.0001
**DBP (mmHg)**	74.13 ± 2.069	102.13 ± 2.352	< 0.0001
**SBP (mmHg)**	120.54 ± 3.859	159.38 ± 2.386	< 0.0001
**Proteinuria (+)**	-	++~+++	N/A

Abbreviation: PE, preeclampsia; NP, normal pregnancy; SPE, severe preeclampsia. All data are presented as means± SEM. N/A = not applicable.

### Expression of *NR5A2* and *NR5A1* in decidual tissues

**Fig 1:**
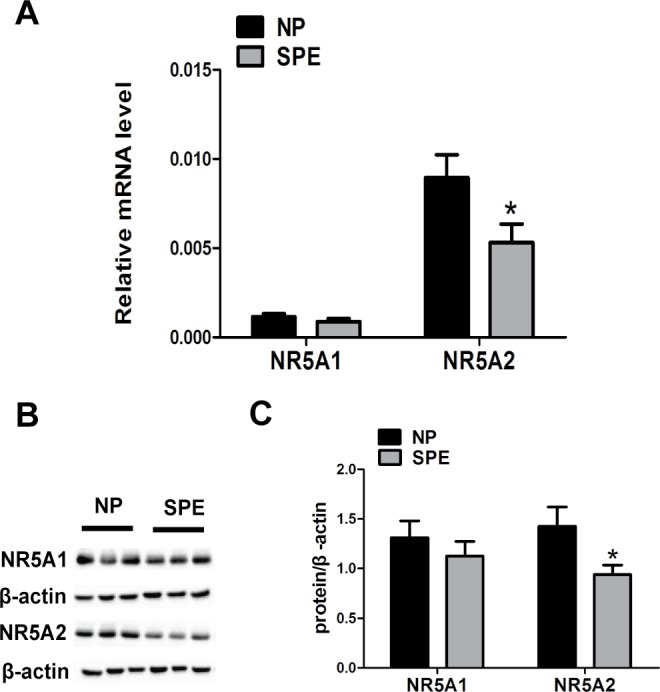
*NR5A1* and *NR5A2* mRNA and protein expression in the decidual tissues of women with and without SPE. (A) The mRNA expression of *NR5A1* and *NR5A2* in the decidual tissues of subjects from the NP and SPE groups. (B) The protein expression of NR5A1 and NR5A2 in the decidual tissues of subjects from the NP and SPE groups. (C) The relative expression levels of the NR5A1 and NR5A2 proteins compared with that of β-actin (*n* = 23 for each group). NP, normal pregnancy group. SPE, severe preeclampsia group. The data were shown as mean ± SEM, *P < 0.05.

**Fig 2:**
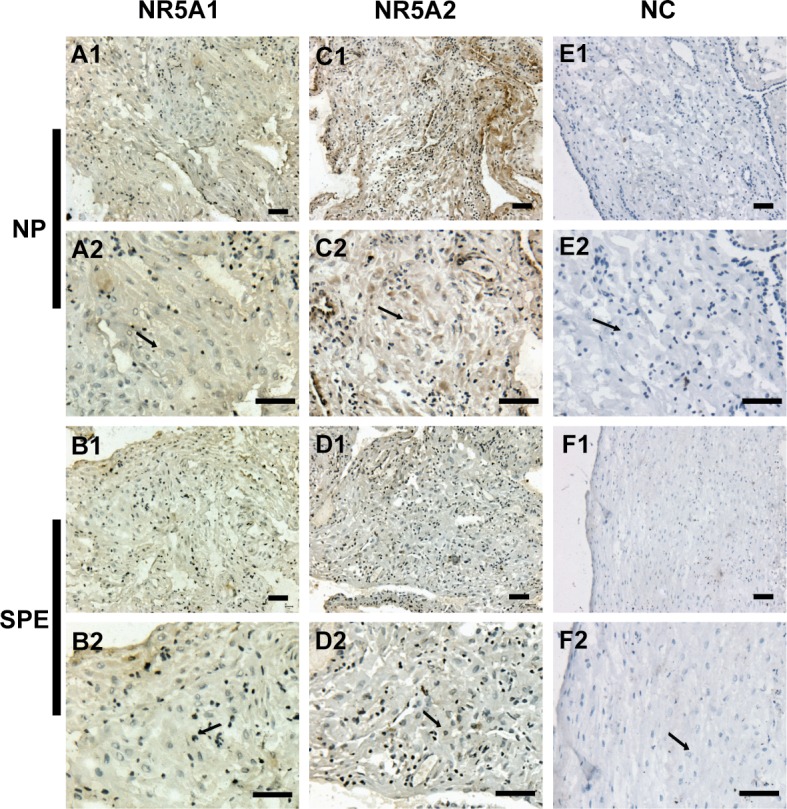
Immunohistochemical expression of NR5A1 and NR5A2 proteins in the decidual tissues of NP and SPE subjects. (A1), (A2), (C1), (C2), (E1), and (E2) depict the decidual sections from the NP group. (B1), (B2), (D1), (D2), (F1), and (F2) were from the SPE group. The brownish particles represent the target protein and the arrows indicate decidual cells. Scale bars = 50 μm. NP, normal pregnancy group. SPE, severe preeclampsia group. NC, negative control.

Decidual tissues from 46 women were examined for the expression of *NR5A1* and *NR5A2*. Of these tissue samples, 23 were obtained from NP women and the rest from women with SPE. The transcribed and translated levels of *NR5A2* were significantly lower in the SPE group than those in the NP group (Fig 1A, 1B and 1C). As for *NR5A1*, there were no differences in the transcribed or translated levels between the two groups (Fig 1A, 1B and 1C). Immunohistochemistry was performed to locate the NR5A1 and NR5A2 proteins (Fig 2). The NR5A1 and NR5A2 proteins were both detected in decidual cells and NR5A2 protein was less abundant in the decidual cells of the SPE group relative to that of the NP group (Fig 2C and 2D). However, there was no difference in NR5A1 protein abundance between the SPE and NP groups (Fig 2A and 2B).

### *NR5A2* is involved in induced decidualization *in vitro*

**Fig 3:**
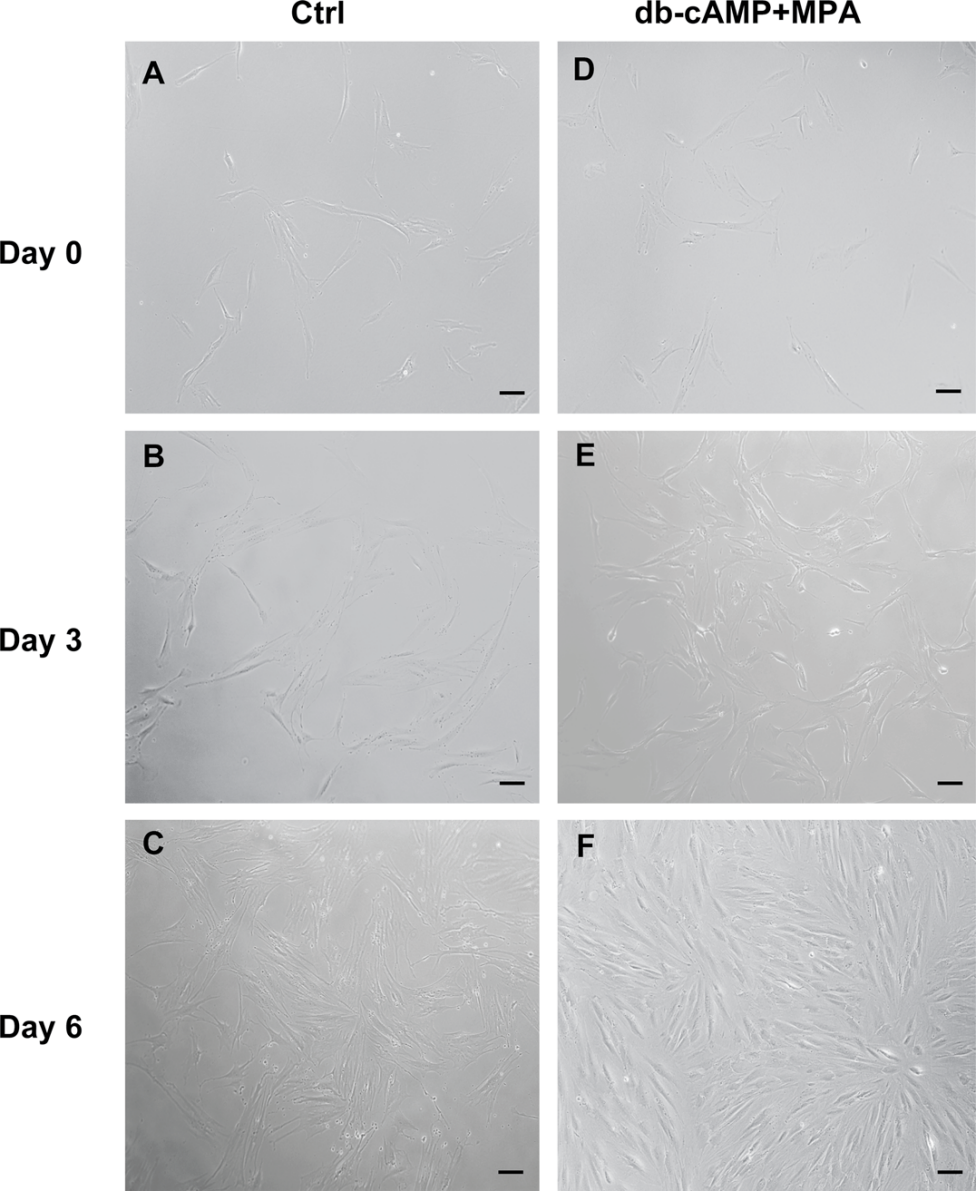
Morphological changes of hESCs in the process of induced decidualization. (A), (B), and (C) show the morphology of control hESCs on days 0, 3, and 6. (D), (E), and (F) show the morphology of the induced hESCs on days 0, 3, and 6. Ctrl, control hESCs; db-cAMP+MPA, treated hESCs. Scale bars = 50 μm.

*In vitro* decidualization of hESCs was performed to investigate the involvement of *NR5A1* and *NR5A2* in this process. Firstly, the morphological changes during the decidualization of hESCs were evaluated *in vitro*. As shown in Fig 3A–3F, hESCs were elongated and had a fibroblast-like phenotype in the control sample (Fig 3E). The cells became rounded, relatively large epithelioid-like or polygonal decidual cells after treatment with db-cAMP and MPA for six days (Fig 3F).

**Fig 4:**
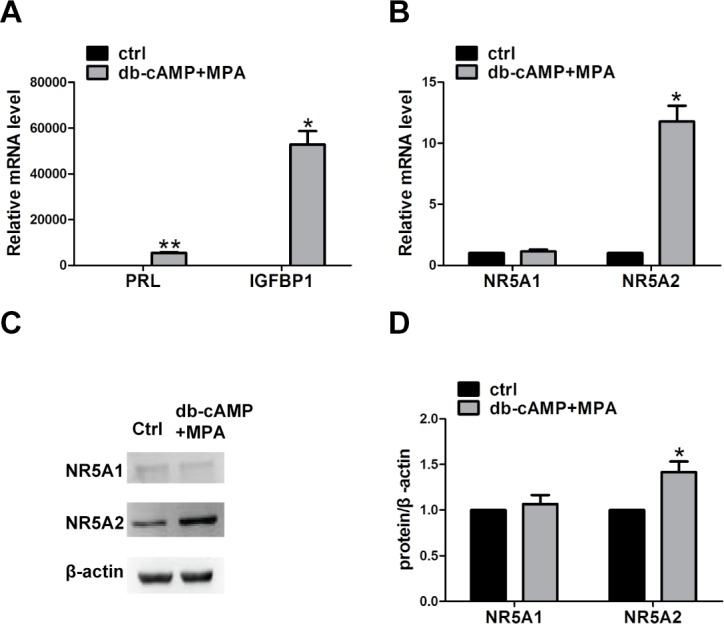
The expression of *NR5A1* and *NR5A2* after the induction of decidualization for 6 days. (A) The mRNA expression of *PRL* and *IGFBP1* after induction of decidualization. (B) The mRNA expression of *NR5A1* and *NR5A2*. (C) Bands representing NR5A1, NR5A2, and β-actin proteins on a western blot. (D) The relative expression levels of NR5A1 and NR5A2 to that of β-actin. The data were shown as mean ± SEM, *n* = 5, *P < 0.05, and **P < 0.01. Ctrl, control hESCs; db-cAMP+MPA, treated hESCs. PRL, prolactin; IGFBP1, insulin-like growth factor binding protein 1; *P < 0.05; **P < 0.01.

The detection of endometrial decidualization biomarkers (*IGFBP1* and *PRL*) mRNA after treatment with db-cAMP and MPA indicated that the *in vitro* induced decidualization system was reliable (Fig 4A). During decidualization, the mRNA expression level of *NR5A2* mRNA level increased by about 11-fold, while the transcription rate of *NR5A1* was low and showed no statistically significant differences compared to that of the controls (Fig 4B). Similar results were demonstrated by western blot analysis (Fig 4C and 4D).

### Knocking down of *NR5A2* affected decidualization

**Fig 5:**
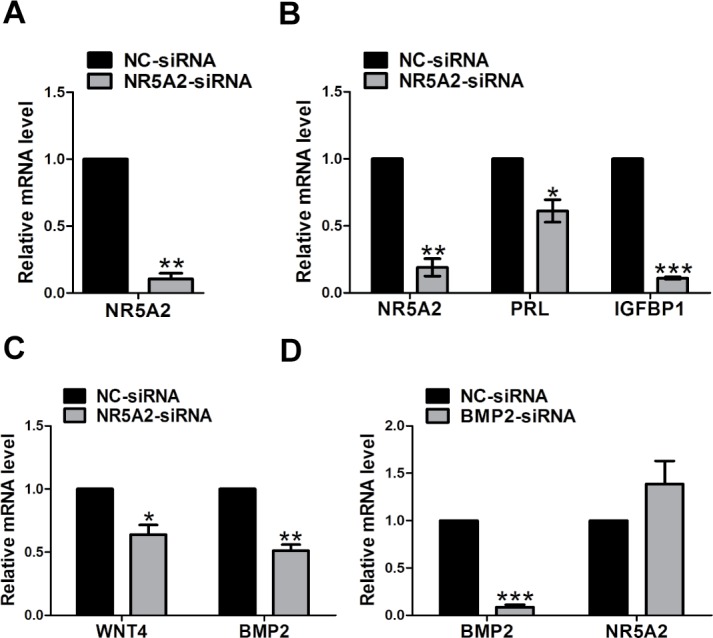
Molecular expression mechanism underlying the effect of *NR5A2* interference. (A) Efficiency of *NR5A2* siRNA mediated knockdown. (B) The mRNA expression of *PRL* and *IGFBP1* after the knocking down of *NR5A2*. (C) The mRNA expression of *WNT4* and *BMP2* after the knocking down of *NR5A2*. (D) The mRNA expression of *BMP2* and *NR5A2* after the knocking down of *BMP2*. The data are expressed as the mean ± SEM. PRL, prolactin; IGFBP1, insulin-like growth factor binding protein 1; *P < 0.05; **P < 0.01; ***P < 0.001.

To further elucidate the function of *NR5A2* during the *in vitro* decidualization of hESCs, the level of *NR5A2* mRNA in the cells was knocked down using RNA interference to silence endogenous *NR5A2* expression. The siRNA targeting *NR5A2* mRNA or a non-targeting siRNA were transfected into hESCs for 24 h and then subjected to differentiation. Fig 5A showed that the hESCs transfected with *NR5A2* siRNA exhibited a reduction of almost 90% in *NR5A2* mRNA expression compared to that of the cells transfected with control siRNA. This down-regulation of *NR5A2* expression in hESCs resulted in a significant reduction in the expression of mRNAs corresponding to *PRL* and *IGFBP1* (Fig 5B).

It has been previously reported that *WNT4* acts downstream of *BMP2* to regulate human endometrial stromal cell differentiation [31] and that *WNT4* is a direct target of *NR5A2* [14]. The expression of *WNT4* in the *NR5A2* silencing system was quantified, and the results demonstrated that *WNT4* mRNA was also down regulated after *NR5A2* mRNA was knocked down (Fig 5C). The relationship between *NR5A2* and *BMP2* during decidualization was explored. As shown in Fig 5C, the mRNA level of *BMP2* was reduced in hESCs treated with *NR5A2* siRNA; however, the *NR5A2* mRNA level was not significantly changed after *BMP2* was knocked down (Fig 5D).

## Discussion

The present study represents a comprehensive investigation of *NR5A1* and *NR5A2* expression levels in PE pregnancies. The roles of both *NR5A1* and *NR5A2* in the decidualization of hESCs were explored and their relationships to PE were inferred. Quantitative real-time PCR and immunohistochemical analyses were used to quantify the gene expression of *NR5A1* and *NR5A2* in the decidual tissue samples of both healthy women and those with SPE. The transcription and translation of *NR5A2* were significantly lower in the decidual tissues of women with SPE compared to those of the women in the gestational matched control group, whereas no significant differences in *NR5A1* expression were observed between the two groups. In the *in vitro* decidualization induction system, there was an apparent change in the cellular morphology of hESCs. In addition, the expression of *NR5A2* was significantly increased at both the RNA and protein levels during decidualization along with the upregulation of the decidualization markers *PRL* and *IGFBP1*, while the levels of *NR5A1* did not significantly change. The knocking down of *NR5A2*, *WNT4*, and *BMP2* using siRNA resulted in significant decreases in the expression of *PRL* and *IGFBP1*. Conversely, after the knocking down of *BMP2*, the transcription of *NR5A2* did not change significantly. These findings demonstrated that *NR5A2* participates in decidualization and that its deficiency in the decidua likely causes PE. As for *NR5A1*, we concluded that it is not involved in human decidualization.

Due to its high occurrence rate and potentially deadly consequences for the mother and/or her offspring, PE has attracted much attention in the medical research community. Even after decades of research, a full understanding of the pathogenesis of PE remains elusive. Currently, the only definitive treatment for PE is to terminate the pregnancy and deliver the placenta and the infant, hence PE accounts for 15% of all preterm births in the United States [32].

PE is a multifactorial disease and its etiological agents include placental, immunological, and maternal factors, among others [33]. Previously, it has been commonly accepted that errors during the development of placenta are the primary source of problems that can lead to PE. However, from the results of this research, it has been reasoned that the failure or deficiency of decidualization may lead to PE, since the decidua can regulate the invasion of EVT cells and protect them against inflammatory responses, which is critical for successful pregnancy.

The involvement of *NR5A2* in the processes of follicular development, female reproduction, and steroidogenesis has been well documented [14, 34, 35]. It has also been reported that *NR5A2* is expressed in the endometrial stromal cells of mice and humans [14]. The deletion of *NR5A2* in mouse uterine stromal cells leads to the phenotype of PE, which has been attributed to a deficiency of decidualization. As expected, there was a significant decrease in the expression of *NR5A2* at the mRNA and protein levels in the decidua of women with PE. Thus, these findings suggest that *NR5A2* may be associated with PE and are consistent with a previous report regarding abnormal decidualization in association with PE [36]. *NR5A1*, also known as steroidogenic factor 1, is another orphan nuclear receptor belonging to the same orphan nuclear receptor family as *NR5A2*. *NR5A1* is closely related to *NR5A2* and these receptors share similarities in their DNA binding domains and DNA response elements [20]. Both NR5A1 and NR5A2 can activate the transcription of genes encoding steroidogenic enzymes [37]. It was predicted from their similar DNA-binding properties and expression patterns that they would have homologous roles during decidualization. Contrary to this hypothesis, there was no significant difference in *NR5A1* mRNA or protein expression between the decidual tissues of subjects from the NP and SPE groups, suggesting that *NR5A1* may be not involved in PE. It is worth mentioning that this is the first report on the expression of *NR5A1* and *NR5A2* in the decidual tissues of PE. The mechanism of their mutual interaction is unclear and merits further study in relation to PE. Our recent study demonstrated that when *NR5A2* is deleted in mouse cells, *NR5A1* cannot compensate for its absence [14].

To further study how the aberrant expression of *NR5A2* may cause PE, decidualization *in vitro* was induced. After decidual stimulation, the morphology of stromal cells changed significantly at 3 and 6 days; the cells and their nuclei became enlarged compared to unstimulated cells. All of the described changes are significant as they closely relate to cell functions. It is known that the decidua can secrete proteins and cytokines such as the decidual markers *PRL* and *IGFBP1*. The expression of *NR5A2* was found to increase markedly at the mRNA and protein levels after decidual stimulation for 6 days. However, the expression of *NR5A1* remained low. It was concluded that *NR5A1* may be not involved in decidualization and is not one of the factors in PE pathogenesis. This *in vitro* induction system further suggested that although *NR5A2* and *NR5A1* belong to the same family and are similar in evolution, there are still differences between them. The results of this study indicated that *NR5A2* was essential for decidualization, which is consistent with our previous work [14].

It has previously been demonstrated by chromatin immunoprecipitation that *WNT4* is a direct target of *NR5A2*, suggesting that *WNT4* is involved in decidualization [14]. It has also been suggested that *WNT4* is the target of *BMP2* in the signaling pathway directing the decidualization process [26]. In addition, previous studies uncovered a direct pathway involving BMP2, WNT4/β-catenin, and forkhead box protein O1 that operates in the human endometrium to control decidualization [13]. The results of this study demonstrated that the expression of *BMP2* and *WNT4* mRNA was reduced after the knocking down of *NR5A2* mRNA, leading to the observed decrease of the decidual markers *PRL* and *IGFBP1* and further demonstrating the significance of *NR5A2* in decidualization. However, after the knocking down of *BMP2* mRNA, the level of *NR5A2* mRNA was not changed. Hence, it is concluded that *NR5A2* may act on *BMP2* to regulate hESC differentiation. Further experiments are needed to confirm this.

In summary, we speculated that *NR5A2* plays an important role in human decidualization by regulating the expression of *WNT4* and *BMP2*. The dysregulation of *NR5A2* in the decidua jeopardized the decidualization process, which in turn damaged the development of the placenta by affecting the invasion of EVT cells into the uterine decidua. These events would consequently harm the establishment of a favorable maternal-fetal connection and could subsequently lead to the occurrence of PE. Normal decidualization is thus essential for normal pregnancy.

## Supporting Information

S1 Table Primer sequences of all genes. (DOCX)

## Abbreviations

PE: preeclampsia
SPE: severe preeclampsia
NP: normal pregnancy
IGFBP1: insulin-like growth factor binding protein 1
PRL: prolactin
EVT: extravillous trophoblastic
SF-1: **NR5A1**, steroidogenic factor-1
LRH-1: **NR5A2**, liver receptor homolog-1
LH: luteinizing hormone
FSH: follicle-stimulating hormone
BMP2: morphogenetic protein
hESC: human endometrial stromal cell
MPA: medroxyprogesterone-17-acetate
cAMP: N6, 2′-*O*-dibutyryladenosine
