# Supplementary material for: Dysfunction of Liver Receptor Homolog-1 in Decidua: Possible Relevance to the Pathogenesis of Preeclampsia
Source: PLoS One. 2015 Dec 30;10(12):e0145968. doi: 10.1371/journal.pone.0145968 (PMC4696807; doi:10.1371/journal.pone.0145968)
Supplement: S1 Table — (DOCX) [file pone.0145968.s001.docx]

| **Gene** | **Primer sequences** | | **Fragment size（bp）** |
| --- | --- | --- | --- |
| *GAPDH* | Forward Primer | 5’- GGAGCGAGATCCCTCCAAAAT-3’ | 197 |
|  | Reverse Primer | 5’- GGCTGTTGTCATACTTCTCATGG-3’ |  |
| *NR5A1* | Forward Primer | 5’- AAGACGCTCAGGAGAAGG-3’ | 155 |
|  | Reverse Primer | 5’- CAGGTGCTTGTGGTACAG-3’ |  |
| *NR5A2* | Forward Primer | 5’- GCGTGGAGGAAGGAATAAGTT-3’ | 224 |
|  | Reverse Primer | 5’- CATAGTCTGTAGGAGGCAAGG-3’ |  |
| *PRL* | Forward Primer | 5’- CATATTGCGATCCTGGAATGAG-3’ | 158 |
|  | Reverse Primer | 5’- GATGAACCTGGCTGACTATCA-3’ |  |
| *IGFBP-1* | Forward Primer | 5’- GGCACAGGAGACATCAGGAGAA-3’ | 131 |
|  | Reverse Primer | 5’- GGTAGACGCACCAGCAGAGT-3’ |  |

**S1 Table. Primer sequences of all genes.**
